# The impact of health insurance on outpatient utilization and expenditure: evidence from one middle-income country using national household survey data

**DOI:** 10.1186/1478-4505-5-6

**Published:** 2007-05-30

**Authors:** Björn Ekman

**Affiliations:** 1 Health Economics Program (HEP), Department of Clinical Sciences, Malmö, Lund University, Sweden

## Abstract

**Background:**

Achieving universal health insurance coverage by means of different types of insurance programs may be a pragmatic and feasible approach. However, the fragmentation of the health financing system may imply costs in terms of varying ability of the insurance programs to improve access to and reduce spending on care across different population groups. This study looks at the effect of different types of health insurance programs on the probability of utilizing care, the intensity of utilization, and individual spending on care in Jordan.

**Methods:**

Using national household survey data collected in 2000 with a sub-sample of around 8,300 individuals, the study applies econometric techniques to a set of specified models along the two-part model approach to the demand for health care. By means of particular tests and other procedures, the robustness of the results is controlled.

**Results:**

Around 60 percent of the population is covered by some type of insurance. However, the distribution varies across income groups, and importantly, the effect of insurance on the outcome indicators differ substantially across the various programs. Generally, insurance is found to increase the intensity of utilization and reduce out-of-pocket spending, while no general insurance effect on the probability of use is found. More specifically, however, these effects are only found for some programs and not for all. The best performing programs are those to which the somewhat better off groups have access.

**Conclusion:**

Notwithstanding the empirical nature of the issues, the results point at the need to assess the effect of insurance coverage more profoundly than what is commonly done. Applying rigorous analysis to survey data in other settings will contribute to bringing out better evidence on what types of programs perform most effectively and equitably in different contexts.

## Background

Health financing systems vary considerably across countries and regions in terms of levels, models and organizational arrangements. While all systems aim at achieving the main financing functions of raising revenues, pooling resources for risk sharing, and purchasing services, models range from universal health insurance coverage by means of a National Health Service (NHS) system or a social insurance program in most high-income OECD-countries, to multiple providers of health care and insurance in many middle-income countries (MICs), and finally, to little prepayment coverage in a range of low- and middle-income countries (L&MICs). While it is safe to suggest that as a result of these variations, outcomes in terms of access to health care (as seen by utilization rates) and individual spending for care vary across health financing systems, too little is known about the particular effects of various health financing systems, specifically for L&MICs [1,2]. These countries frequently display highly fragmented financing systems with possibly adverse effects on access and personal spending. This study aims at contributing to filling this gap in the current body of evidence by applying rigorous quantitative methods to household level data from Jordan to analyze the effect of multiple health insurance availability in terms of utilization of outpatient care, intensity of use, and individual out-of-pocket (OOP) spending on care. Specifically, the study assesses whether insurance programs differ as to the impact on utilization and spending, and if the impact vary across income groups.

The specific context and challenges for middle-income countries have recently been discussed [[1], ch. 8]. Among other things, it is noted that as levels of health spending have reached around 5–6 percent of GDP (or, approximately, US$ 80–100 per capita per year), current health financing policies aim at providing universal coverage of health services with a focus on equity in health financing and efficiency in service provision. Health insurance is a key instrument in current health financing reforms in many MICs and it is noted that these countries may need to use a combination of different insurance schemes to reach the goals of universal coverage and financial protection. As is further discussed below, Jordan is in many ways a case in point: around 10 percent of GDP is spent on health with multiple insurance options co-existing, while some 40 percent of the population is without any financial protection mechanism.

Although there are several important benefits of financing health care through insurance, the existence of a fragmented health insurance system may lead to both inefficiencies and equity concerns. These concerns may be both direct and indirect and affect the overall health system as well as the individual patient. Indirectly, an ineffective and inequitable financing system may be a source of social discontent, particularly in a society that spends a large share on care. An inefficient health protection system may have an impact on the ability of the health system to achieve its overall policy objectives of health financing and financial protection. Given the particular data that are used in this study, a close assessment of the larger social impacts of the Jordanian health insurance system is beyond the scope of this paper.

This paper is organized as follows. In the remainder of this section, a brief discussion of the theory of the demand for health care and the role of insurance is provided followed by a description of some key dimensions of the Jordan health financing system. Section two describes the data and the methods of the study and section three presents the findings. Section four discusses the results and the final section provides some conclusions of the study in terms of policy and further analysis.

### Theory and review of the literature

From a health economics perspective, there are, in principle, two alternative views on the demand for health care. One suggestion is that the individual demands care as an input into her production of health [3]. This view – sometimes referred to as the Grossman model – suggests that the demand for health care is a derived demand in the process of investment in health capital. The Grossman model of the demand for health thus views health care as an input along with other health inputs such as nutrition and personal exercise. Specifically, the model views the individual as the sole decision maker as to if and how much health care to use.

Over the past decade, however, the Grossman model of the demand for health has been challenged by a complementary view that sees the demand for health care within a principal-agent framework [4]. In this view, the individual decides if and when to seek health care while the provider of the services decides how much care to use once the first decision has been taken. One of the criticisms of the Grossman model is the fact that many of its predictions are not supported by the empirical analyses; see, among others, [5] for a recent discussion of these issues. Depending on the particular view of the demand for health care that one adopts, the methods for analyzing the effect of for instance health insurance on the demand for care will vary. This is further discussed in the Methods section below.

The role of insurance in health financing is twofold, one to raise revenues for health care services, and two, to pool these resources so that health risks can be effectively shared among the members of the insurance scheme [6]. Given the uncertainty with which ill health affects a given individual in the population, risk sharing is both an equitable and an effective way of financing health care. Indeed, important policy outcomes of health insurance are to improve access to care and to reduce individual spending at the time of use, which is particularly important for those with limited ability to pay. By bringing the direct price of health care down, consumption of care will increase, all else considered. However, the extent to which this occurs in any given context is an empirical issue, given other factors, such as indirect costs, that may still impede access to care by some groups of the population (other problems with health insurance are moral hazard and adverse selection, but these are not the subject of analysis in this paper).

Based on these theoretical underpinnings, the effect of health insurance on utilization and expenditures has been analyzed in different ways in the empirical literature. As suggested, the general task has been to choose an appropriate model to deal with the dual decision process of the demand for health care, i.e. the first decision to see a health care provider, and then, the second decision of how much care to obtain [7,8]. The principal focus of the sizable literature on health insurance has been the U.S. health care market with its highly fragmented third-party payment structure, involving both public and private programs while at the same time leaving some 16 percent of the population without health insurance at any given time [6]. The best known health insurance study is the Rand Health Insurance Experiment (HIE) [9]. The randomized design of the HIE provided a unique opportunity to study the effect of insurance on the health care seeking behavior of individuals. The RAND study finds, among other things, evidence for increased health spending when insurance coverage is complete, compared with incomplete coverage.

Another early contribution uses household level data from Australia to look at two things [10]. First, the authors develop a microeconomic model for individual choice under uncertainty for various health insurance programs and health care types. They find that utilization choices vary considerably across insurance types, but that health status proves to be a stronger determinant of utilization of care than for insurance choice. And second, they find evidence of both self-selection and moral hazard due to insurance for some health care types although not for all.

In recent years, a small but growing number of studies have looked at the effect of health insurance on access and expenditures in low- and middle-income countries. For Vietnam, one study looks at the effects of voluntary health insurance on the choice of provider and type of care [11]. Using instrumental variable (IV; to control for the possible endogenous nature of insurance status) multinomial logit estimations for a sample of 1,800 individuals and controlling for, among other factors, individual health status, the authors find evidence of moral hazard, as poorer insured persons tend to use inpatient care more compared with poorer uninsured individuals, a difference that is not found at higher income levels. In another study on Vietnam the effect of public voluntary health insurance on private expenditures is analyzed [12]. Here, the authors use a version of the Heckman sample selection bias model to correct for possible endogeneity of the health insurance status dummy variable. For a sample of 980 individuals, the authors find evidence of an insurance protection effect while controlling for a set of exogenous explanatory variables including health status and income.

For a South American country, one study uses probit estimation techniques to study the effect of health insurance on utilization of curative and preventive care in Ecuador [13]. Correcting for potential endogeneity the author finds that in a sample of around 3,300 individuals, insurance has a positive effect on the utilization of curative care, but only a small effect on preventive care. Finally, the effect of a national school health insurance program on utilization and expenditures in Egypt is studied [14]. Using a two-step estimation approach in a sample of 17,000 eligible schoolchildren, the findings suggest that on average the program increases access to care and reduces financial outlays.

Although the above studies find a positive insurance effect on average, the effect is not uniform across income groups. To assess the role of insurance across various groups, all of the above studies (with the possible exception of [13]) use insurance/income interaction terms. The sign of these variables suggests that this effect varies across income groups. For example, insurance is found to reduce health expenditures more for the poor than for the rich in Vietnam [12]. In Egypt, only the middle-class children benefited from a reduced financial burden due to insurance. However, it is unclear whether these interaction effects are computed correctly as the true effect of an interaction term in a logit or probit estimation may differ, both in sign and magnitude as well as in statistical significance, from the one reported by the initial estimation command [15,16]. In the statistical software program Stata (used here), a special **'inteff' **command can be invoked to correctly calculate the interaction effect [16]. Moreover, only one of the above studies [10] looks at the effect of more than two insurance programs simultaneously. Thus, this study contributes to the existing evidence base by analyzing multiple insurance in a middle-income country context and by correctly considering the income interaction effect.

### The Jordanian context

Jordan is a lower middle-income country situated in the Middle-East with a gross national income (GNI) that is slightly above that of its regional neighbors [17]. However, annual economic growth in recent years has been limited at around 1.5 percent per person. In terms of overall health expenditure, Jordan spends considerably more than do its immediate neighbors- with the exception of Lebanon – and other MICs: around 9 percent of GDP compared with 5 percent and 6 percent, respectively. Almost half of all health care spending is private in the form of insurance premiums and as direct out-of-pocket payments at the time of need. Public spending on health is mostly directed towards inpatient services with only around a quarter spent on outpatient care. The single most expensive part of health care in Jordan is pharmaceuticals that account for one third of total spending. In combination with changing demographic and epidemiological patterns, it is suggested that the country will find it difficult to sustain such high levels of health spending without undertaking health financing reform efforts with the overall aim of providing effective care while protecting the population from catastrophic consequences when seeking care [18,19].

Several actors provide health care services in Jordan, the largest of which is the Ministry of Health (MoH). Other providers include the Royal Military Services (RMS), Jordan University Hospital (JUH), and the private sector. Along with the United Nations' regional refugee program (UNRWA), these are also the main providers of health insurance, to which around 60 percent of the population in Jordan has access. Insurance coverage is higher among women, elderly, illiterate, and rural population groups. Of those with insurance, around 11 percent has multiple insurance coverage, which is less than anticipated prior to the current survey. See table 1 for more details on, among other things, insurance coverage.

**Table 1:** Descriptive statistics

**Variable/Indicator**	**Frequency**	**Mean/Share (%)**	**Min**	**Max**	**S.D.**
**Background indicator(s):**					

Illness in past 14 days*	1,632	19.65	0	1	-

**Dependent variables:**					

Outpatient visit (if ill)*	1,034	63.3	0	1	-
Intensity of utilization: *			0	1	
• One visit (if visit)	887	86	0	1	-
• Two visits (if one visit)	123	12	0	1	-
• Three visits (if one visit)	18	1.74	0	1	-
• Four visits (if one visit)	4	0.39	0	1	-
• Five visits (if one visit)	1	0.1	0	1	-
• Six visits (if one visit)	1	0.1	0	1	-
Out-of-pocket expenditure (total per episode; current JD)	-	5.31	0	400	0.53

**Policy variable:**					

Insured*	5,328	64	0	1	-
• MoH	1,514	28	0	1	-
• RMS	2,312	43	0	1	-
• JUH	68	1	0	1	-
• UNRWA	668	13	0	1	-
• Private	764	14	0	1	-

**Control variables:**					

SES – total household expenditure (current JD)	-	222	10	2200	168.26
Age (years)		25.72	0	98	20.31
Male*	4,059	48.87	0	1	-
Education (Completed school years; adults)	-	10.66	1	25	3.44
Marital status*	3,014	36.29	0	1	-
Labor status: paid employee*	2,001	24,09	0	1	-
Health status:*					
• Regular visit to health care provider for chronic illness	1,487	17.9	0	1	
• Regular medication	1,192	14.34	0	1	-
• Self-assessed health: Better	1,962	23.62	0	1	-
• Self-assessed health: Similar	5,696	68.58	0	1	-
• Self-assessed health: Worse	648	7.8	0	1	-
• North*	2,349	28.28	0	1	-
• Central*	4,113	49.52	0	1	-
• South*	1,844	22.20	0	1	-
Urban*	5,967	71.84	0	1	-
Own house*	5,533	66.61	0	1	-

Full sample (random individuals)	8,306	-	-	-	-

Source: JHES 2000. - = not applicable. * = binary indicator variable.

Separate agreements among the various non-private insurance programs enable transferability. For example, anyone insured by MoH can be transferred to the JUH; in emergency cases such services would be free of charge. The Ministry of Health's insurance program, Civil Insurance Program (CIP), covers several groups of people, including public civil servants and their dependants, government retirees and their families, beneficiaries of the National Aid Fund, blood donors, and also the poor, the handicapped, and others who are able to pay the premiums. This program provides treatment free of charge for a list of diseases and health conditions, including cancer, dialysis, anemia, and other blood-related diseases.

The JUH insurance program covers University employees and their families, employees of other universities, and staff of certain large companies according to special agreements with the University Hospital. The Royal Medical Services (RMS) insurance program covers the following categories of individuals: current and retired staff of the united armed forces, members of the royal court, telecom company staff and family members, and a variety of members of other organizations and entities in the society. Lastly, UNRWA provides care for around 400,000 Palestinian refugees, most of which is maternal and child health care. Members of UNRWA can be transferred to a government hospital through a special agreement with the Ministry of Health.

## Data and methods

The present study uses data from the Jordan Healthcare Utilization and Expenditure Survey (JHUES) collected in 2000. The survey contained a sample of 8,800 households and obtained information on each individual within the household, the head of the household, and detailed health care utilization and expenditure information on a randomly selected household member. Essentially, the JHUES consists of four samples: (i) demographic and background data on 49,543 individuals, (ii) insurance coverage on 49,456 individuals, (iii) household data on 8,306 heads of households, and (iv) health care utilization and spending data, and self-assessed health (SAH) on 8,306 randomly selected individuals of all ages. Individuals from this latter sub-sample are used in the subsequent analysis.

The survey instrument was divided into seven sections: the household schedule, health insurance data, outpatient care utilization (two-week reference period), health status, inpatient episodes, mortality, and household conditions, including income and assets. Moreover, the survey used a complex sample design with seven strata. In the first stage of the sampling method, the primary sampling units (psu) were selected within each stratum with a probability proportional to size (pps). In the second stage, 20 households were randomly selected within each sampling unit. Finally, as noted, one individual was chosen at random within each household. Sampling weights were then calculated in two steps. First, each observation was weighted by the inverse of its probability of selection, and second, the weights were normalized by dividing the first step probability weights by their average. The sample design and sampling weights are considered in all estimates in the ensuing analysis; this ensures robust standard errors of all estimated coefficients.

Descriptive statistics for the main variables are presented in table 1. During the recall period of two weeks prior to the survey, a little less than 20 percent of the randomly interviewed individuals reported having had at least one spell of illness. Out of those individuals, around 63 percent visited a health care provider for outpatient care, the vast majority of which made only one visit and only a handful made more than three visits.

As previously noted, around 64 percent of the Jordanian population has access to some type of health insurance. The most common insurance program is the Royal Medical Services (RMS), which provides health insurance for 43 percent (28 percent of all randomly interviewed individuals; not shown) of the insured. The program organized by the Ministry of Health (CIP) provides insurance for another 28 percent (18 percent) of the insured while the Jordanian University Hospital (JUH) program only reaches a little more than one percent (less than 1 percent) of the insured individuals. Some 14 percent (9 percent) of those with insurance have some form of private health insurance, and finally, the UN special program for the Palestinian refugees in Jordan (UNRWA) provide health insurance for around 13 percent (8 percent) of all those insured.

Information on health insurance status is provided in two places in the questionnaire: first, for all individuals in the survey (n = 49,456) regardless of possible health care utilization during the recall period, and second, for all of the randomly interviewed persons given that they visited a health care service provider at least once during the recall period. Below the first source of information is used so as to be able to gauge the effect of insurance on utilization regardless of whether a provider was visited or not during illness. Moreover, the Jordan survey collected information on health care utilization for specific illnesses, as opposed to any illness in the recall period. This means that the potential problem of independence between illness and utilization is taken care of, which, in turn, speaks in favor of applying the two-part approach to the analysis of health care demand [7].

### Econometric considerations

In the subsequent analysis, there are at least three specific econometric considerations that require special attention. First, as mentioned earlier, using interaction terms in logit and probit models calls for the application of extended estimation commands in some standard econometric software programs, including Stata [15,16].

Second, the coefficients of the various explanatory variables may be biased and inconsistent if some of them correlate with the error term in the econometric models below, suggesting that they are endogenous. In particular, the health insurance status indicator variable may be suspected of being endogenous due to unobserved heterogeneity if individuals self-select into their insurance status on account of some factor not controlled for and thus contained in the error term in the estimation models [See, for example, [20]].

There are several factors that are not immediately observed why individuals may opt to buy or obtain (a specific type of) health insurance. For example, to the extent that obtaining insurance is costly, richer individuals may choose to buy insurance expecting to utilize health care in the future. Also, health insurance may be associated with a certain type of employment either in the private or the public (including military) sectors. Individuals may then opt to seek certain types of jobs partly with a view to obtaining health insurance in anticipation of future health care needs. Finally, people with poorer health may choose to obtain health insurance due to higher expectations of utilizing health care.

By including measures of these and other factors in the models, attempts are made to control for the potential endogeneity of the insurance variable. In addition, a test for endogeneity of the insurance variable is performed using a version of the Durbin-Wu-Hausman class of tests for endogeneity [21]. Failure to reject the null hypothesis of no correlation between the error term and any of the regressors in such a test would suggest that some of the regressors are endogenous, which, in turn, would suggest a failure to properly identify the regression equation. One way of handling endogeneity would be to use instrumental variable (IV) estimation techniques. While obtaining valid instruments for the endogenous variables is no easy task, and using poor instruments may be inferior to using the possibly endogenous variable and accounting for bias, some instruments that have been suggested in the literature on the demand for health and insurance include the relationship of the individual to the head of household and mean rate of affiliation of the insurance type in the community [13]. This type of information is available from the JHES 2000 survey, although whether they provide valid instruments in this case needs to be tested formally.

The final issue concerns the applicability of the count data model. While a Poisson distribution may be assumed in count data on the number of visits to a provider, the actual estimation of the count model by means of Poisson estimation methods may be inappropriate due to the rather restrictive assumptions of the Poisson model. In particular, the model assumes that the mean of the expected number of counts (or, events) is equal to the variance. Frequently in empirical situations, this assumption is known to be violated due to overdispersion. A formal test of overdispersion may be applied, the outcome of which may suggest an alternative estimation method, such as a negative binomial (negbin) approach [20,21]. Also, the Poisson model assumes that visits are independent, which clearly is a strong assumption given that referrals most likely require the active decision of a GP or similar provider. Finally, the count data on the number of visits to an outpatient provider is truncated, which will have additional implications for the choice of estimation method [20].

### Econometric models

The current set of analyses attempt to assess the effect of insurance on health care utilization and on subsequent payments for care by means of econometric estimation of three separately specified models as outlined below. The key variable of interest is thus 'insurance status', which is a dummy variable taking the value 1 if the individual has health insurance and 0 otherwise. To further gauge the insurance effect, the study allows for the interaction between insurance status and an indicator of individual (or household) socioeconomic status. Here, total household monthly consumption expenditure divided by the square root of household size to get a per capita measure and control for household size, is taken as that indicator.

Based on theory and empirical experience, insurance status is expected to improve access to care and to reduce payments for services. While the data generating process (DGP) underlying these decisions is admittedly complex, the econometric approach essentially depends on the nature of the data and the information they contain about the dependent variables. This issue has been discussed in the literature [22], and a taxonomy to help choose the correct model has been suggested. Based on the discussion, this study adopts the approach of the 'two-part model' (TPM) to assess the effect of insurance on utilization and expenditures.

Formally, the econometric models are the following, where the individual subscript *i *= *(1,...,N) *is suppressed for notational simplicity. The probability of a health care visit conditional on being ill is estimated by the probability model

Prob(utilization>0|ill) = *βX*+*ε*

where *X *is a set of covariates, including health insurance status. This model is linear in the log of the odds (logit) of the event, in this case visiting a provider when ill.

The intensity of health care utilization is analyzed by means of a 0-truncated negative binomial (0-negbin) model since the focus of analysis is the number of visits conditional on at least one visit to a service provider. The negative binomial takes the following general form:

Pr⁡(y|x)=Γ(y+α−1)y!Γ(α−1)(α−1α−1+μ)α−1(μα−1+μ)y
 MathType@MTEF@5@5@+=feaafiart1ev1aaatCvAUfKttLearuWrP9MDH5MBPbIqV92AaeXatLxBI9gBaebbnrfifHhDYfgasaacH8akY=wiFfYdH8Gipec8Eeeu0xXdbba9frFj0=OqFfea0dXdd9vqai=hGuQ8kuc9pgc9s8qqaq=dirpe0xb9q8qiLsFr0=vr0=vr0dc8meaabaqaciaacaGaaeqabaqabeGadaaakeaacyGGqbaucqGGYbGCcqGGOaakcqWG5bqEcqGG8baFcqWG4baEcqGGPaqkcqGH9aqpdaWcaaqaaiabfo5ahjabcIcaOiabdMha5jabgUcaRGGaciab=f7aHnaaCaaaleqabaGaeyOeI0IaeGymaedaaOGaeiykaKcabaGaemyEaKNaeiyiaeIaeu4KdCKaeiikaGIae8xSde2aaWbaaSqabeaacqGHsislcqaIXaqmaaGccqGGPaqkaaGaeiikaGYaaSqaaSqaaiab=f7aHnaaCaaameqabaGaeyOeI0IaeGymaedaaaWcbaGae8xSde2aaWbaaWqabeaacqGHsislcqaIXaqmaaWccqGHRaWkcqWF8oqBaaGccqGGPaqkdaahaaWcbeqaaiab=f7aHnaaCaaameqabaGaeyOeI0IaeGymaedaaaaakiabcIcaOmaaleaaleaacqWF8oqBaeaacqWFXoqydaahaaadbeqaaiabgkHiTiabigdaXaaaliabgUcaRiab=X7aTbaakiabcMcaPmaaCaaaleqabaGaemyEaKhaaaaa@63D7@

where y is a health care visit ranging from one to, in this case, six visits, and Γ(·) is the gamma function.

Finally, out-of-pocket expenditures conditional on positive utilization of outpatient care is estimated by OLS of the log-linear model

Log(OOP|utilization > 0) = *γX*+*μ*

Again, X is a set of explanatory variables, including insurance status. In models (1) and (3), *β *and *γ *are the coefficients to be estimated and *ε *and *μ *are error terms; in model (2), *μ *is the expected number of counts or, here, outpatient visits.

In order to be able to draw a ceteris paribus conclusion as to the insurance effect, the study controls for a number of individual, household, and community factors. The individual factors include age and gender, educational, civil and occupational status, and nationality. There are few convincing a priori reasons to expect these variables to affect utilization and payments in any special direction as these effects are largely empirical. The study does, however, also control for the health status of the individual and it is expected that, all other things being equal, poorer health status leads to larger health care needs and possibly larger health care expenditures.

Household factors include income (as measured by consumption expenditure) and other living conditions of the household. Although these factors are suggested to influence the dependent variables in models (1) – (3), the expected sign of the estimated coefficients of these variables is indecisive, again due to the empirical nature of these effects. Community factors include the presence of a health care provider nearby and a regional dummy variable. Living close to a health service provider is expected to increase the probability of utilization.

## Results

This section presents the findings from the two sets of quantitative analyses. The first part presents the results from looking at the bivariate relationships between the key variables. The findings from this exercise will provide an initial indication of the effect of insurance on utilization and spending on outpatient care. The second part presents the findings from the econometric estimation of models (1) – (3) presented above.

### Bivariate analysis: distribution and relationship of insurance, health, and utilization by SES

It was previously noted that overall insurance coverage in Jordan is around 60 percent of the population. Further analysis of the data suggests that being insured varies by socioeconomic status. For example, in the poorest income quintile, 45 percent do not have health insurance compared with 32 percent in the richest groups (table 2). These differences are statistically significant.

**Table 2:** Tabulation of income and insurance status

**Income quintile**						
	**1**	**2**	**3**	**4**	**5**	**Total**

**Insured?**						
**No**	1,023	571	672	315	397	2,978
	34.35	19.17	22.57	10.58	13.33	100.00
	44.67	32.55	32.67	32.54	32.09	35.85
**Yes**	1,267	1,183	1,385	653	840	5,328
	23.78	22.20	25.99	12.26	15.77	100.00
	55.33	67.45	67.33	67.46	67.91	64.15
**Total**	2,290	1,754	2,057	968	1,237	8,306
	27.57	21.12	24.77	11.65	14.89	100.00
	100	100	100	100	100	100

Pearson chi-squared(4) = 107.03, Pr = 0.00

Source: JHUES 2000

Moreover, the various income groups in Jordan have access to different types of insurance programs (table 3). The poorest group predominantly relies on the RMS, MoH, and the UNRWA programs, and only very few numbers of poor have access to private insurance or the JUH insurance program. In contrast, the richest 20 percent of the population with access to health insurance rely on MoH, private insurance, and RMS. In addition, some 42 percent of all those insured by JUH belong to the richest SES quintile. The findings that insurance status and type of insurance vary across socioeconomic status are statistically significant at the 1 percent level.

**Table 3:** Tabulation of income and type of insurance, Jordan 2000

**Income quintile**						
	**1**	**2**	**3**	**4**	**5**	**Total**

**Insurance type**						
**MoH-CIP**	395	291	378	189	261	1,514
	26.09	19.22	24.97	12.48	17.24	100.00
	31.18	24.60	27.31	28.94	31.11	28.43
**RMS**	542	643	625	255	247	2,312
	23.44	27.81	27.03	11.03	10.68	100.00
	42.78	54.35	45.16	39.05	29.44	43.41
**JUH**	7	6	11	16	28	68
	10.29	8.82	16.18	23.53	41.18	100.00
	0.55	0.51	0.79	2.45	3.34	1.28
**UNRWA**	260	150	158	56	44	668
	38.92	22.46	23.65	8.38	6.59	100.00
	20.52	12.68	11.42	8.58	5.24	12.54
**Private**	63	93	212	137	259	764
	8.25	12.17	27.75	17.93	33.90	100.00
	4.97	7.86	15.32	20.98	30.87	14.34
**Total**	1,267	1,183	1,384	653	839	5,326
	23.79	22.21	25.99	12.26	15.75	100.00
	100	100	100	100	100	100

Pearson chi-square(16) = 537.19, Pr = 0.00

Source: JUHES 2000

Insurance status also varies across health. However, there is no clear pattern in this regard. For example, the hypothesis that self-assessed health is independent from insurance cannot be rejected at the 10 percent significance level (not shown). On the other hand, reported illness is associated with insurance status (p = 0.01), such that those with insurance have a higher probability of reporting an illness. This is in itself an interesting finding that might suggest some process of moral hazard-like behaviour among the insured. Further analysis of this would, however, require more detailed data than are available in the current survey.

Regarding the bivariate relationships between health insurance (and type of insurance) and utilization of care and OOP spending on care, the following is noted. There is no evidence that utilization is associated with insurance status when looking at the sub-sample that reported an illness during the recall period. There is, however, some indication that utilization vary by type of health insurance (not shown). In particular, utilization seems to be associated with access to the CIP program and to private health insurance (p-values = 0.08 and 0.06, respectively), but not to the other types of insurance programs. Furthermore, there is no indication in the data that the intensity of use of outpatient services varies by insurance status or by type of health insurance. There are indications that utilization of outpatient care varies by insurance status for the poorest income group, but not for the more well-off groups.

Health status seems to vary across the income groups. For example, self-assessed health (SAH) and reported illness during the recall period both vary statistically significantly across income groups. Among those who consider themselves in worse health than their peers, 43 percent belong to the poorest income group compared with only 10 percent from the richest group. Conversely, 17 percent of those who consider themselves in better health than their matching group belong to the poorest income group compared with more than 30 percent from the richest group. Similarly, of those who reported being ill during the recall period, some 63 percent belong to the three poorest groups compared with around 33 percent from the two richest groups (table 4).

**Table 4:** Tabulation of income and reported illness, Jordan 2000

**Income quintile**						
	**1**	**2**	**3**	**4**	**5**	**Total**

**Illness in past 2 weeks?**						
**No reported illness**	1,831	1,375	1,632	807	1,029	6,674
	27.43	20.60	24.45	12.09	15.42	100.00
	79.96	78.39	79.34	83.37	83.19	80.35
**Reported illness**	459	379	425	161	208	1,632
	28.13	23.22	26.04	9.87	12.75	100.00
	20.04	21.61	20.66	16.63	16.81	19.65
**Total**	2,290	1,754	2,057	968	1,237	8,306
	27.57	21.12	24.77	11.65	14.89	100.00
	100	100	100	100	100	100

Pearson chi-square(4) = 17.7, Pr = 0.00

Source: JUHES 2000

Finally, the data show that out-of-pocket spending varies across health insurance types with those with access to private health insurance paying the most on average and those with RMS insurance paying the least (not shown). Furthermore, there is evidence in the data that poorer groups spend on average less compared with richer groups (not shown). On the other hand, looking at OOP spending across income groups for the insured and the non-insured, it seems the "insurance effect" (ratio between mean OOP spending of non-insured over insured) is largest in the higher income groups as the insured in these groups seem to spend relatively less than the uninsured compared with the poorer groups.

In summary, this section has shown that insurance coverage and its effect on utilization and spending is far from even across socioeconomic status in Jordan. The further exploration of the insurance effect, however, requires that other factors that also affect utilization and OOP payments are controlled for. This is done in the next section.

### Multivariate analysis: utilization, intensity of use, and OOP spending

Table 5 presents the estimation results of model (1), probability of health care utilization conditional on illness in the past two weeks prior to the survey. Column (1) shows the preferred base model after a series of restrictions has been imposed on a selection of the explanatory variables. Generally, neither income nor insurance significantly increase the probability of making a visit to a health service provider when ill. Instead, the model shows that health status is the most important factor determining outpatient use as those in worse health, as indicated by the self-assessed health indicator and the presence of a chronic illness significantly increase health care utilization. Notably, findings suggest that people increasingly seek less care as they grow older. Compared with those in the central part of the country, those in the north utilize less care. There is no indication that sex or education determines the use of health services. The specified models only explain a share of the variation in health care utilization as indicated by relatively low R-squared values (not shown).

Column (2) performs the same analysis using a disaggregated health insurance status indicator. This exercise shows that the initial result discussed above does not correctly explain the effect of insurance on outpatient use in Jordan. Rather, the disaggregated indicator shows that those with access to the Ministry of Health insurance program CIP have a significantly higher probability of seeking outpatient care than others. As expected, the coefficients of the other explanatory variables remain largely unchanged.

The third column shows that the insurance-income interaction variable is significantly negative suggesting that, as income increases insurance becomes a gradually less important factor to explain utilization of outpatient care. The interaction effects are largely confirmed by the use of the '**inteff'- **command in Stata (these results are not shown for reasons of space, but are available from the author upon request). The effect is considerably more varied, however, for people having a probability of visiting an outpatient provider of around 0.2 compared with a higher probability. Moreover, the true interaction effect does not differ significantly from zero for the sample of individuals in the middle of the probability spectrum. Finally, we learn that the mean interaction effect is -0.05 with a standard error of 0.02 (z statistic is -1.57), i.e. somewhat larger in absolute terms than initially shown in table 5.

**Table 5:** Estimation of model (1) Probability of outpatient use, Jordan 2000

**Dependent variable: Visited outpatient provider when ill**	**(1)**	**(2)**	**(3)**
INCOME	0.125	0.127	0.345**
	[0.12]	[0.12]	[0.15]
INSURED	0.158		1.763**
	[0.14]		[0.83]
AGE	-0.0533***	-0.0537***	-0.0282**
	[0.020]	[0.019]	[0.014]
AGE^2	0.000490*	0.000496*	0.000214
	[0.00026]	[0.00026]	[0.00018]
MALE	-0.127	-0.119	0.0481
	[0.16]	[0.16]	[0.13]
JORDANIAN	-0.224	-0.231	-0.327
	[0.36]	[0.36]	[0.30]
EDUCATION	0.0159	0.0157	-0.00979
	[0.020]	[0.020]	[0.014]
MARRIED	0.339*	0.338*	0.168
	[0.19]	[0.19]	[0.16]
PAID EMPLOYEE	0.215	0.229	0.176
	[0.21]	[0.21]	[0.18]
SAH-similar	0.200	0.198	0.172
	[0.16]	[0.16]	[0.13]
SAH-worse	0.434*	0.430*	0.425**
	[0.25]	[0.25]	[0.20]
CHRONIC ILLNESS	0.665***	0.664***	0.638***
	[0.25]	[0.25]	[0.22]
DISABLED > 3 days	0.682***	0.683***	0.630***
	[0.25]	[0.24]	[0.17]
CENTRAL REGION	-0.101	-0.0950	-0.0601
	[0.15]	[0.15]	[0.14]
NORTH REGION	-0.530***	-0.532***	-0.541***
	[0.15]	[0.15]	[0.14]
MOH insurance		0.341*	
		[0.19]	
RMS insurance		0.119	
		[0.16]	
JUH insurance		-0.0906	
		[0.64]	
UNRWA		0.229	
		[0.23]	
PRIVATE insurance		0.165	
		[0.26]	
INC*INSURANCE			-0.344*
			[0.19]
Constant	0.579	0.532	-0.531
	[0.62]	[0.62]	[0.72]
Observations	1632	1632	1632

Robust standard errors in brackets. * 10%, **5%, ***1%

Results from the analysis of the impact of health insurance on the intensity of health care utilization, model (2) are presented in table 6. While the interpretation of the coefficients of the count data model is relatively straightforward [see, for example, [23], for details], the focus here is principally on the sign of the coefficients. Column (1) shows that having access to some type of health insurance significantly increases utilization of health care compared with being exempted to pay for care.

**Table 6:** Estimation of model (2): Intensity of outpatient use, Jordan 2000

**Dependent variable: Total number of visits conditional on at least one visit**	**(1)**	**(2)**	**(3)**
INCOME	-0.170	-0.181	-0.327
	[0.16]	[0.17]	[0.28]
INSURED	0.342*		-0.631
	[0.20]		[1.44]
AGE	0.00183	0.00179	0.00225
	[0.022]	[0.022]	[0.022]
AGE^2	-0.000108	-0.000123	-0.000109
	[0.00030]	[0.00030]	[0.00030]
MALE	-0.238	-0.224	-0.231
	[0.20]	[0.20]	[0.20]
JORDANIAN	-0.264	-0.367	-0.274
	[0.42]	[0.43]	[0.42]
EDUCATION	0.0268	0.0313	0.0257
	[0.023]	[0.023]	[0.023]
MARRIED	0.491*	0.476*	0.487*
	[0.26]	[0.25]	[0.25]
PAID EMPLOYEE	-0.501*	-0.496*	-0.495*
	[0.27]	[0.27]	[0.27]
SAH-similar	0.771***	0.775***	0.763***
	[0.28]	[0.28]	[0.28]
SAH-worse	0.995***	1.009***	0.988***
	[0.33]	[0.33]	[0.33]
CHRONIC ILLNESS	0.408	0.407	0.399
	[0.29]	[0.29]	[0.29]
DISABLED > 3 days	0.658***	0.660***	0.653***
	[0.20]	[0.20]	[0.20]
NEAR PROVIDER	0.0506	-0.0439	0.0408
	[0.23]	[0.24]	[0.23]
CENTRAL	0.385*	0.454**	0.399*
	[0.21]	[0.22]	[0.21]
NORTH	0.00959	-0.00774	0.0126
	[0.24]	[0.24]	[0.24]
MOH insurance		0.385*	
		[0.22]	
RMS insurance		0.574***	
		[0.22]	
JUH insurance		-0.808	
		[1.07]	
UNRWA		0.0862	
		[0.30]	
PRIVATE insurance		0.0724	
		[0.32]	
INC*INSURANCE			0.228
			[0.33]
Constant	-2.182**	-2.002**	-1.492
	[0.98]	[0.99]	[1.39]

lnAlpha	0.0181	-0.0894	-0.00445
	[1.02]	[1.00]	[1.01]
Observations	1034	1034	1034

Robust standard errors in brackets. * 10%, **5%, ***1%

In addition, compared with those with better self-assessed health status, those with similar and worse health use more care, as does having a more serious illness. While these results are in line with expectations, it might also be noted that proximity to a provider does not significantly determine intensity of outpatient use. Age does not explain intensity of use, while being married and living in the central part of the country significantly increases outpatient utilization. Finally, being a paid employee significantly reduces the number of outpatient visits per illness episode.

The results shown in column (2) indicate that the insurance effect differs significantly across insurance programs. The MoH and the RMS programs significantly increase the number of visits to a provider when ill. This effect is not seen in any of the other included insurance programs. In line with the results in the previous sub-section, the findings suggest that access to care varies by insurance affiliation. Column (3) shows that in contrast to the finding in model (1), the income-insurance interaction term is not statistically significant.

Table 7 shows the effects of health insurance on levels of health care spending, model (3). As in the previous analyses, column one shows the base model with the aggregate health insurance dummy variable. The results suggest that, generally, health insurance in Jordan significantly reduces levels of health spending on outpatient care. The results also indicate that those with higher incomes, live near a provider, reside in the central part of the country, and have worse health pay more for care compared with their respective counter groups.

**Table 7:** Estimation of model (3): Outpatient care expenditures, Jordan 2000

**Dependent variable: Total OOP spending per episode**	**(1)**	**(2)**	**(3)**	**(4)**
INCOME	2.298***	2.363***	3.941***	
	[0.52]	[0.54]	[0.97]	
INSURED	-2.649***		9.821**	-2.657***
	[0.62]		[4.22]	[0.64]
AGE	-0.0278	-0.0400	-0.0246	-0.0547
	[0.075]	[0.075]	[0.075]	[0.073]
AGE^2	0.000542	0.000727	0.000480	0.000912
	[0.0011]	[0.0011]	[0.0011]	[0.0011]
MALE	0.312	0.255	0.176	0.410
	[0.52]	[0.52]	[0.52]	[0.52]
JORDANIAN	-2.867	-2.498	-2.784	-2.697
	[2.67]	[2.74]	[2.66]	[2.65]
EDUCATION	0.143	0.156	0.140	0.180*
	[0.11]	[0.11]	[0.10]	[0.11]
MARRIED	0.194	0.296	0.292	0.353
	[0.76]	[0.76]	[0.76]	[0.77]
PAID EMPLOYEE	-0.0656	-0.162	-0.214	-0.0212
	[1.04]	[1.04]	[1.07]	[1.04]
SAH-similar	1.864***	1.809***	1.846***	1.729***
	[0.52]	[0.52]	[0.52]	[0.51]
SAH-worse	0.478	0.327	0.549	0.0192
	[0.75]	[0.73]	[0.74]	[0.75]
CHRONIC ILLNESS	3.825*	3.892*	3.917*	3.837*
	[2.19]	[2.20]	[2.20]	[2.18]
DISABLED > 3 days	3.254**	3.215**	3.263**	3.223**
	[1.31]	[1.30]	[1.31]	[1.30]
NEAR PROVIDER	3.213***	3.329***	3.268***	3.157***
	[0.56]	[0.57]	[0.56]	[0.56]
CENTRAL	1.797***	1.590***	1.651***	1.943***
	[0.43]	[0.47]	[0.42]	[0.45]
NORTH	-0.681	-0.684	-0.730*	-0.700*
	[0.42]	[0.44]	[0.43]	[0.42]
MOH insurance		-2.934***		
		[0.57]		
RMS insurance		-2.802***		
		[0.57]		
JUH insurance		-2.353		
		[1.75]		
UNRWA		-0.844		
		[0.79]		
PRIVATE insurance		-1.371		
		[1.32]		
INC*INSURANCE			-2.867***	
			[1.05]	
Tertial richest				-2.443***
				[0.83]
Tertial middle				-1.348
				[0.95]
Constant	-7.420**	-7.953**	-14.51***	3.919
	[3.02]	[3.13]	[4.38]	[2.67]

Observations	8306	8306	8306	8306
R-squared	0.11	0.11	0.11	0.10

Robust standard errors in brackets. * 10%, ** 5%, ***1%

Column (2) shows the disaggregated insurance effect. As with the above analyzes, the true effect of insurance depends on the particular type of program. Specifically, those with access to the CIP and RMS programs pay significantly less than those with no insurance, while this effect is not found for the other programs.

The third column of table 7 shows that the income-insurance interaction term is significantly negative, suggesting that it is the more well-off groups that benefit from the financial protection effect of having insurance. In line with this result, the last column shows the results of dividing the households into three groups based on income. This suggests that compared with the poorest households, higher income groups pay significantly less for outpatient care.

## Discussion

This paper analyzes the impact of various types of health insurance programs on outpatient health care utilization and subsequent spending on care in Jordan with a specific focus on effects across socioeconomic status. Applying specific quantitative techniques to individual level survey data, the study has been able to reveal evidence that the effect of health insurance on these outcomes varies by type of insurance and socioeconomic status. This section discusses these findings and the validity of the results, starting with some methodological considerations.

## Methods

A general point is the reference to the principal-agent framework in the beginning of the paper. The methodological approach has been largely built on an assumption that the individual decides on the initial choice of care and the provider decides on subsequent utilization of care, single-handedly or in collaboration with the patient. The role of insurance has then been the particular objective of the study. A specific issue to note is the relatively low overall explanatory power of these econometric models. The fact that they only explain a share of the determinants of the outcomes is not surprising given the cross-sectional nature of the data [24]. On the other hand, the rigorous application of suitable econometric models that are in turn based on theory and empirical experience hopefully ensures that the explanatory power of the analysis sufficiently increases the validity of the results to be of actual policy relevance.

Furthermore, the above models have been subjected to a number of specification and diagnostic tests. In particular, the possible endogeneity of health insurance in models (1) and (3) has been tested by means of the aforementioned Durbin-Wu-Hausman (DWH) test. The insurance variable (*hins*) would be endogenous in, for example, model (1) if Cov(*hins,ε*) differs from 0, where *hins *∈ *X *in (1) and (3). This would render the coefficient of *hins *biased and inconsistent. The test is performed as follows. First, regress *hins *on all other exogenous variables, including the selected IVs (in this case, government affiliation, work status, and Palestinian). Obtain the reduced form residual terms, say, ν⌢
 MathType@MTEF@5@5@+=feaafiart1ev1aaatCvAUfKttLearuWrP9MDH5MBPbIqV92AaeXatLxBI9gBaebbnrfifHhDYfgasaacH8akY=wiFfYdH8Gipec8Eeeu0xXdbba9frFj0=OqFfea0dXdd9vqai=hGuQ8kuc9pgc9s8qqaq=dirpe0xb9q8qiLsFr0=vr0=vr0dc8meaabaqaciaacaGaaeqabaqabeGadaaakeaacuaH9oGBgaWeaaaa@2E7E@. Include ν⌢
 MathType@MTEF@5@5@+=feaafiart1ev1aaatCvAUfKttLearuWrP9MDH5MBPbIqV92AaeXatLxBI9gBaebbnrfifHhDYfgasaacH8akY=wiFfYdH8Gipec8Eeeu0xXdbba9frFj0=OqFfea0dXdd9vqai=hGuQ8kuc9pgc9s8qqaq=dirpe0xb9q8qiLsFr0=vr0=vr0dc8meaabaqaciaacaGaaeqabaqabeGadaaakeaacuaH9oGBgaWeaaaa@2E7E@ in (1) and formally test if the coefficient for ν⌢
 MathType@MTEF@5@5@+=feaafiart1ev1aaatCvAUfKttLearuWrP9MDH5MBPbIqV92AaeXatLxBI9gBaebbnrfifHhDYfgasaacH8akY=wiFfYdH8Gipec8Eeeu0xXdbba9frFj0=OqFfea0dXdd9vqai=hGuQ8kuc9pgc9s8qqaq=dirpe0xb9q8qiLsFr0=vr0=vr0dc8meaabaqaciaacaGaaeqabaqabeGadaaakeaacuaH9oGBgaWeaaaa@2E7E@ is statistically significant from zero (H0: β_hins_resM _= 0). Failure to reject the null hypothesis would suggest that *hins *is exogenous in (1). The test indicates that health insurance is not endogenous in model (1); chi2(1) = 0.01, prob(chi2) = 0.93.

In addition, residual analysis (specifically the Cook's Distance statistica) suggested that a handful of observations might be suspected of being outliers, with large influence on the final estimation results. However, repeating the analysis while excluding these suspected observations did not in any way change the results.

Finally, all of the model estimations were bootstrapped to obtain an indication of the robustness of the results. The findings of this analysis suggested that the estimated coefficients only have small biases; differences were found in the fourth or fifth decimal place.

### Utilization of and spending on outpatient care

All three models illustrate the main point of this analysis, that the true impact of insurance may be hidden when only looking at the aggregate effect. Specifically, model (1) shows that, in general, health insurance does not affect the probability of utilizing health care. However, the true effect of insurance is revealed by looking at the specific programs. It is then shown that the Civil Insurance Program significantly increases the probability of seeking care when ill (p < 0.1).

In terms of the intensity of outpatient care, the various insurance programs in Jordan have very different effects. While the analysis showed that insurance in general significantly increased the number of visits per illness episode, the large insurance programs run by the Ministry of Health and the Royal Medical Services largely explained this result. The other programs did not affect the number of outpatient visits.

The effects of health insurance on health care expenditures is somewhat mixed in the above analysis. Generally, there is a suggestion that overall spending is reduced by insurance. However, looking across table 7 shows that this result is not robust across alternative specifications and analyses suggesting that further assessment may be warranted to shed light on this issue.

Several important factors may explain many of the findings. One obvious factor that drives the results is the heterogeneity of the Jordanian health financing system and, in particular, the compartmentalization of the various financing schemes. The very existence of these different programs and an inability to effectively overcome this nature of the health financing system are reflected in the results and, importantly, in the current reform aims of the government of Jordan [18,19]. As pointed out by one commentator of this paper, recent innovations by the Royal Court – a centrally funded program to assist, in particular, poor people with no other insurance option – will clearly contribute to increasing the real coverage rate of the country and newer assessments using more recent data may be able to pick this up. In the current analysis, any impact of this program is picked up by controlling for income.

Obtaining a more unified system will most likely contribute to reducing these varying effects across socioeconomic groups and insurance types. In this process, there are, of course, several alternative options, one of which is to obtain a more coherent legislative framework so that the different programs are obliged to cover some level of minimum care, while simultaneously allowing increased transferability between programs. In addition, although it is beyond the ability of this study to say with any certainty, it is possible that such a health financing system would have favorable social impacts by contributing to generating a coherent social security arrangement.

## Conclusion

As countries are trying to achieve universal coverage of health care services to its citizens by means of increasing prepayment programs, reform efforts will most likely make use of several types of insurance programs, both compulsory and voluntary. While such approaches can be seen as the most pragmatic given economic and institutional constraints to the ability to rapidly expand government programs, this study has shown that these processes may come at some cost in terms of socioeconomic inequality as different insurance programs are associated with varying ability to provide access to care and financial protection.

The empirical nature of these issues suggests at least three implications that need to be taken into consideration. First, the results from this study are likely to have small external validity, which, in turn, suggests the second point, that similar analysis should be undertaken in other settings. The combined implications of these two points is that once a sufficiently large number of similar studies have been undertaken that looks systematically at the particular effects of various health insurance types, systematic reviews can be done to draw more general conclusions as to the most preferred models of health insurance in various settings.

Finally, in terms of policy, this study has highlighted the need for analysts and decision makers to be adamant of the efficiency and equity implications of trying to achieve universal coverage of health services by means of health insurance. Continuous monitoring and rigorous evaluation of the potentially varying real coverage of existing policy instruments will remain at the top of the health reform agenda in low- and middle-income countries for many years to come.

## Competing interests

The author(s) declare that they have no competing interests.

## Authors' contributions

The author is fully responsible for all parts of the study.
